# Short-term primary and revision modular dual-mobility cup total hip arthroplasty outcomes in high-risk dislocation patients: a retrospective study

**DOI:** 10.1007/s00590-024-04092-5

**Published:** 2024-09-08

**Authors:** Martta Ruusiala, Hannu Miettinen, Jukka Kettunen, Heikki Kröger, Simo Miettinen

**Affiliations:** 1 Department of Orthopaedics, Traumatology and Hand Surgery, Kuopio University Hospital, P.O. Box 1777, 70211 Kuopio, Finland; 2 Faculty of Health Sciences, University of Eastern Finland, Yliopistonranta 1, 70210 Kuopio, Finland

**Keywords:** Total hip arthroplasty, Dual-mobility cup, Survival, Complications, Revision

## Abstract

**Purpose:**

Modular dual-mobility cups (MDMCs) have a lower risk for dislocation after total hip arthroplasty (THA). The primary aims of our study were to analyze implant survivorship and to determine complications, especially dislocation, and revision rates of primary THAs used for hip fracture patients and for revision THAs. Secondary aims were to evaluate mortality after MDMC surgery and to find out if introduction of MDMC at our institution (Kuopio University Hospital, Finland) have decreased dislocation rate.

**Methods:**

This retrospective cohort study consisted of 101 MDMC patients who were consecutively operated at our institution between April 1, 2018 and June 30, 2020. The implant survival rate, complications and mortality were evaluated with minimum of 2-year follow-up. Finnish Hospital Discharge Register was used to find out yearly dislocation rates following THA at our institution.

**Results:**

The cumulative estimate implant survival after MDMC in the primary THA group was 97% at 2 years, and in the revision THA group, it was 90% at 2 years. Dislocation was a rare complication in the primary THA group (1.4%), while it was common in revision THA group (12.9%). The cumulative estimate for mortality after MDMC in the primary THA group was 13% at 2 years, and in the revision group, it was also 13% at 2 years. The yearly number of patients who had re-hospitalization period due to THA dislocation decreased 46% after implementation of MDMC.

**Conclusion:**

Short-term survival and complication rates after MDMC were excellent after primary THA and moderate after revision THA. Implementation of MDMC THA for hip fracture patients seems to have effectively decrease dislocation rate during a short follow-up.

## Introduction

Total hip arthroplasty (THA) is an effective surgical procedure to enhance patient’s ability to move, quality of life and reduce pain [1]. THA is the indicated treatment for primary osteoarthrosis (OA), hip fractures, rheumatoid arthritis (RA), avascular necrosis (AVN) of the femoral head, posttraumatic conditions, hip dysplasia, and post-arthritis conditions [2, 3]. Common risk factors for THA failure and revision surgery are age > 75 years, female gender and acetabulum component position out of Lewinnek safe zone (5°–25° anteversion and 30°–50° inclination) [4–8]. When one or more of these known risk factors are present, the patient should be considered at high-risk of complications. However, hip instability following hip arthroplasty is possibly the leading cause of failure [4–7]. Well-known risk factors for dislocation after THA are a small femoral head size (< 32 mm), operation due to femoral neck fracture (FNF), post-traumatic arthrosis, AVN of the femoral head, hip dysplasia, obesity (body mass index [BMI] > 30 kg/m^2^), excessive alcohol consumption, and degenerative neurological diseases (e.g., Alzheimer’s disease, Parkinson’s disease, and dementia) [9, 10].

In the 1990s and early 2000s, there was an attempt to avoid dislocations by using larger head sizes with metal-on-metal (MoM) bearings, but this usage has decreased due to complications like adverse reaction to metal debris associated with severe osteolysis and bone loss [11–13]. Since then, dual-mobility cup (DMC) and modular dual-mobility cup (MDMC) THAs have gained popularity. They represent a good option to avoid dislocations in hip fracture patients associated with high-risk of dislocation [7, 9, 10]. The MDMC was introduced in France more than 40 years ago and has been increasingly used in revision as well as primary THAs with various indications, but widespread international adoption has been much slower probably due to insufficiency of long-term register data, cost-burden and different variation of complications compared to conventional THAs [7, 9, 10, 14–16]. Currently, long-term outcome data for MDMC implants are limited to the French experience, and no long-term data are available [16].

MDMC implant is designed to increase hip stability by providing large-head articulation and increased jumping distance. MDMC implants can contribute to a high range of motion of the hip joint and reduce the risk of dislocation [7, 17, 18]. MDMC implants have a lower risk for revision due to dislocation compared with conventional THAs. The dislocation rate after primary MDMC THA is between 0 and 10%, while after conventional THA it is 0.5–22% [7, 10, 19–21].

In this retrospective study, we present a single university hospital experience with MDMC THA for high-risk patients with 2-year follow-up. The primary aims of our study were to analyze implant survivorship and to determine complications and reoperation rates of primary and revision THAs. The secondary aims were to evaluate mortality after MDMC surgery and to evaluate if introduction of MDMC at our institution have decreased dislocation rate of THAs. We hypothesized that dislocation complications are rare in both MDMC groups and the number of patients treated for dislocation following THA have decreased after implementation of these implants at our institution in 2018.

## Materials and methods

### Setting, participants, and implants

This was a retrospective cohort study of consecutive cases performed at a single institution (Kuopio University Hospital, Finland). A total of 101 patients who had primary (*n* = 70/101 ([70%]) or revision (*n* = 31/101 [30%]) THA at a university teaching hospital between April 1, 2018, and June 30, 2020, were included. A minimum of 2 years of follow-up was set, ending on June 30, 2022, or following the death of the patient. At our institution, we are used to perform THA rather than hemiarthroplasty for (a) active patients who were independently mobile before a displaced FNF and for (b) patients who have other well-known risk factors (e.g., hip dysplasia, post-traumatic arthrosis, AVN, or degenerative neurological disease) for dislocation. These 101 MDMCs THAs were the first ones operated at our institution and none of the patients were excluded from this study.

We evaluated how introduction of MDMC at our institution in year 2018 has affected the yearly number of hip dislocations following THA. The annual number THA patients treated for dislocation at our institution was harvested from the Finnish Hospital Discharge Register (FHDR) provided by the Finnish Institute for Health and Welfare [22]. From FHDR data, we screened the annual number of all primary THA patients operated at our institution and (a) who had undergone primary THA due to any reason (including hip fracture), (b) who had undergone THA with any kind of implant due to hip fracture and (c) had a hospitalization period due to hip dislocation during one-year follow-up after implantation of primary THA between years 2008 and 2020.

Patient data, including age, gender, operation side, operation indication, and such as surgical details, were collected from the hospital’s medical records. Complications were defined as minor (no need for revision) and major (revision surgery or other serious adverse event). The major complications were defined as dislocation, prosthetic joint infection (PJI), loosening of the implant, periprosthetic fracture, nerve damage, and other serious events. Major complications with and without revision surgery were analyzed separately. Other complications, like superficial infection, were considered minor. The time from the primary surgery to complication was evaluated.

All patients received a hemispherical MDMC implant (Novae® E TH, SERF, Décines-Charpieu, France). An acetabular titanium shell with a cementless or cemented porous interface was used, and the mean diameter of the MDMC was 51 (range 29–62) mm in both groups (Table 1). The metallic head size was either 22 or 28 mm. The femur stem was cemented in 57/101 (56%) of the cases (Table 1). The femur components were Lubinus SPII® (Link, Hamburg, Germany), Summit® (Johnson & Johnson, Warsaw, Indiana, USA), Taperloc® (Biomet, Warsaw, Indiana, USA), Biomet Reach® (Biomet, Warsaw, Indiana, USA), Corail® (Johnson & Johnson, Warsaw, Indiana, USA), and Spectron® (Smith & Nephew, Watford, UK). In one revision case, the stem was changed due to loosening in addition to revision of the acetabulum component.

**Table 1:** Patients’ demographic characteristic

	Primary THA	Revision	
	(*n* = 70)	(*n* = 31)	*p*-value
	*n* (%)	*n* (%)	
Gender			0.50^✢^
Female	39 (56)	15 (48)	
Male	31 (44)	16 (52)	
Operation indication			< 0.001^✢^
Hip fracture	47 (67)	0	
Primary osteoarthrosis	11 (16)	0	
Dislocation	0	13 (42)	
Component instability	0	12 (39)	
Avascular necrosis	7 (10)	0	
Infection	0	4 (13)	
Post-traumatic arthrosis	4 (6)	0	
Pain	0	2 (6)	
Hip dysplasia	1 (1)	0	
Operation side			0.31^✢^
Left	37 (53)	13 (42)	
Right	33 (47)	18 (58)	
Surgical approach			
Posterolateral	68 (97)	27 (87)	
Lateral hardinge	2 (3)	4 (13)	
Acetabulum component fixation			< 0.001^✢^
Cementless	50 (71)	7 (23)	
Cemented	20 (29)	24 (77)	
MDM metallic head size			0.02^✢^
22 mm	2 (3)	5 (16)	
28 mm	68 (97)	26 (84)	
Radiological analysis	Mean (SD, range)	Mean (SD, range)	
Acetabulum component position			
Inclination angle°	48 (9, 31–71)	47 (9, 27–68)	0.73^◇^
Anteversion angle°	23 (9, 4–43)	19 (9, 1–35)	0.03^◇^
Acetabular component position at Lewinnek safe zone*			0.41^✢^
Yes	31 (44)	11 (65)	
No	39 (56)	20 (35)	

*THA*, total hip arthroplasty; *MDMC*, modular dual mobility cup

*Lewinnek safe zone (5–25° anteversion and 30–50° inclination) described by Lewinnek et al. [8]

^✢^Chi squared test, ^◇^Independent samples *t*-test

### Surgical technique and follow-up

The operations were performed by consultant orthopedic surgeons or by orthopedic residents with supervision of a consultant orthopedic. Overall, 64/101 (63%) of the operations were done by four experienced consultant orthopedic surgeons. All the revision cases were performed by these four experienced consultant orthopedic surgeons. The posterolateral approach was used in all cases. Spinal anesthesia was given for 76/101 (76%) of the patients; the remaining patients (25/101, 24%) received general anesthesia. A single dose of antibiotic prophylaxis (cefuroxime 3.0 g or clindamycin 900 mg) was given preoperatively and routine postoperatively anticoagulation was prescribed for 4 weeks in all cases. Free range of motion and full-weight bearing was allowed immediately after operation for all patients. A routine outpatient follow-up visit was scheduled 3 months after THA.

Retrospective radiological evaluation was done by authors M.R. and S.M. from the radiographs taken at the 3 months postoperative control. Inclination and anteversion of the acetabulum component were measured from anteroposterior (AP) and mediolateral (M/L) native plain radiographs. The evaluation was carried out using a picture archiving and communication system (PACS).

### Statistical analysis

Kaplan–Meier survivorship analysis was performed to calculate survivorship and freedom from revision for any reason. Categorical variables are expressed as frequencies and percentages, and continuous variables are presented as the mean and standard deviation (SD). Continuous data were compared using the Mann–Whitney U-test. Categorical data were compared with the Chi-square test and *t*-test. Cox regression analysis was used to evaluate common risk factors for revision (age [≤ 75 and > 75 years], gender, acetabulum component position [Lewinnek safe zone, 5°–25° anteversion and 30°–50° inclination] [8], and operation type [primary or revision]). Fisher’s exact test was used to analyze operation diagnosis as a risk factor for revision. The hazard ratios (HR) with their respective 95% confidence intervals (CI) were specified for each estimate of the model parameters. A *p*-value < 0.05 were considered statistically significant. The data was analyzed using SPSS Statistics Version 27.0 (IBM Corp., Armonk, NY, USA).

## Results

### Patients and demographics

The study cohort comprised 70 primary THAs and 31 revision THAs (101 patients). The mean follow-up time was 2.4 years (SD 9.9 months, range 5 days to 3.8 years). The mean age of the patients was 71.4 (SD 10.3, range 42.9–88.3) years in the primary THA group and 75.1 (SD 8.8, range 60.6–89.3) years in the revision THA group. The demographic characteristics of the patients are given in Table 1.

**Fig. 1:**
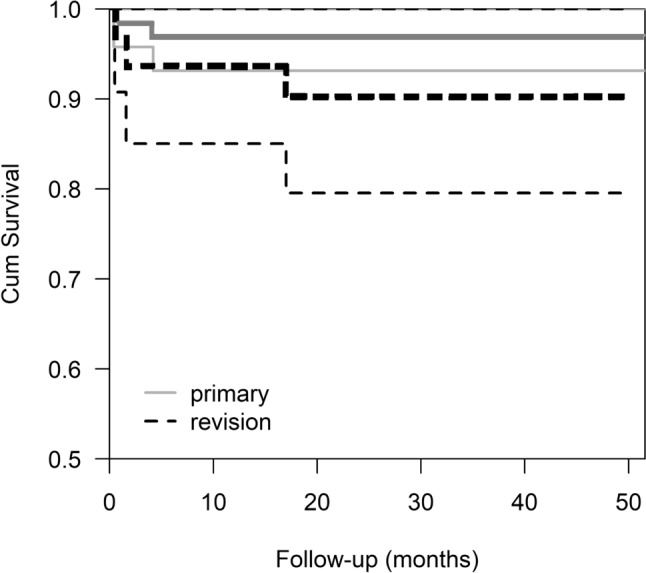
Kaplan–Meier survival analysis for time. The cumulative estimate for survival with no need for revision surgery after MDMC in the primary surgery group was 97% at 1 year, at 2 years and at 3 years (SE 1.0, CI 95% 48.2–52.1), and in the revision surgery group, it was 93% at 1 year and 90% at 2 years and at 3 years (SE 2.4, CI 95% 40.8–50.4) (Log rank test, *p* = 0.15)

According to FHDR data, the annual number of hip fracture patients treated with THA increased 25% after the introduction of MDMC in 2018 at our institution. Between the years 2008 and 2017, the mean annual number of these patients was 29.6 (range, 22–40) and since 2018, the mean annual number was 40.7 (range, 39–42) (Fig. 1). Between the years 2008 and 2017, the mean annual number of the patients revised due to THA dislocation was 23.9 (range, 18–38), and after the year 2018, it was 13 (range, 10–17) (Fig. 1). The annual number of patients who had undergone primary THA due to any reason and had later during one-year follow-up a hospitalization period due to dislocation decreased 46% after introduction of MDMC.

### Complications and reoperations

A total of 18/101 (18%) complications occurred and of these, 15/18 (61%) were major and 3/18 (39%) were minor (Table 2). The cumulative estimate for survival with no need for revision surgery after MDMC in the primary surgery group was 97% at 1 year, at 2 years and at 3 years (SE 1.0, CI 95% 48.2–52.1), and in the revision surgery group, it was 93% at 1 year and 90% at 2 years and at 3 years (SE 2.4, CI 95% 40.8–50.4) (Fig. 1). The mean time from the index surgery to revision was 0.7 years (SD 0.9, range 12 days to 1.3 years) years in the primary THA group and 29 days (SD 18, range 14–49 days) in the revision THA group (*p* = 0.30).

**Table 2:** The number of major complications and reoperations after modular dual mobility total hip arthroplasty

	Primary arthroplasty (*n* = 70)		Revision arthroplasty (*n* = 31)		*p*-value†
	Complications	Of which resulted in revision	Complications	Of which resulted in revision	
	*n* (%)	*n* (%)	*n* (%)	*n* (%)	
*Major complication type*					
Dislocation	1 (1.4)	0	4 (12.9)	1 (25)	0.03
Periprosthetic joint infection	1 (1.4)	1 (100)	5 (16.1)	5 (100)	0.01
Loosening of the acetabulum component	1 (1.4)	1 (100)	1 (3.2)	1 (100)	1.00
Periprosthetic fracture	2 (2.8)	1 (50)	0	0	0.57

^†^Chi-squared test

### Risk factor analyses for reoperations

The Cox regression analysis showed that age, gender, acetabulum position at the safe zone, and operation type (primary vs. revision) were not significant risk factors for revision surgery after MDMC THA (Table 3). Fisher’s exact test showed that of the operation diagnose AVN was associated to risk of reoperation in the primary THA group, and dislocation, infection, and component instability were associated with risk of reoperation in the revision THA group (*p* = 0.03).

**Table 3:** Cox-regression analysis for revision risk factors

	*n* (%)	HR	95% CI	*p*-value
*Age*				
< 75 years	53 (52)	1.00 (Ref.)		
≥ 75 years	48 (48)	0.76	0.13–4.53	0.76
*Gender*				
Male	47 (47)	1.00 (Ref)		
Female	54 (53)	1.33	0.22–7.97	0.75
*Operation type*				
Primary	70 (69)	1.00 (Ref)		
Revision	31 (31)	3.44	0.58–20.61	0.18
Acetabular component position at Lewinnek safe zone*				
No	59 (58)	1.00 (Ref.)		
Yes	42 (42)	0.34	0.04–3.03	0.33

*CI*, confidence interval; HR, hazard ratio

*Lewinnek safe zone (5–25° anteversion and 30–50° inclination) described by Lewinnek et al. [8]

### Mortality

**Fig. 2:**
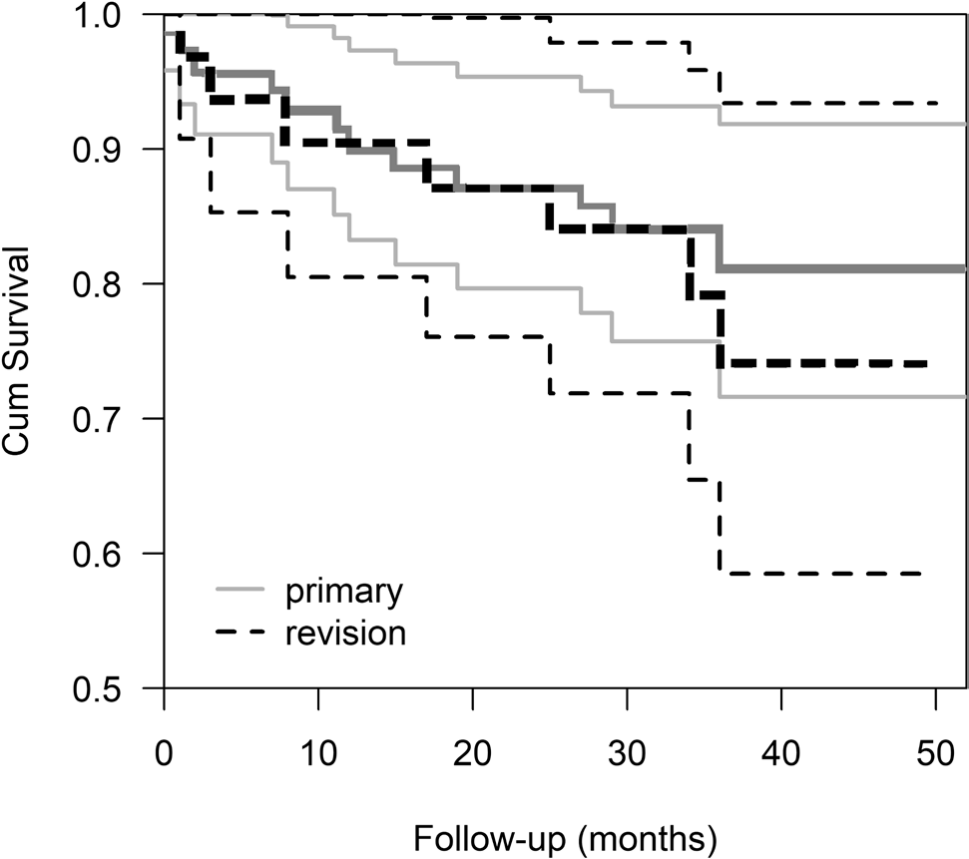
Kaplan–Meier survival analysis of time. The cumulative estimate for mortality after MDMC THA in the primary surgery group was 10% at 1 year, 13% at 2 years and 19% at 3 years (SE 189, CI 95% 41.6–48.8), and in the revision group, it was 10% at 1 year, 13% at 2 years and 26% at 3 years (SE 2.7, CI95% 36.8–47.6) (Log rank test, *p* = 0.60)

The cumulative estimate for mortality after MDMC THA in the primary surgery group was 10% at 1 year, 13% at 2 years and 19% at 3 years (SE 189, CI 95% 41.6–48.8), and in the revision group, it was 10% at 1 year, 13% at 2 years and 26% at 3 years (SE 2.7, CI95% 36.8–47.6) (Fig. 2). A total of 19/101 (19%) of the patients died due to any reason during follow-up at different time points prior to end of the 2-year follow-up.

## Discussion

DMC THAs have shown good overall survivorship after primary and revision THA [23]. In their meta-analysis of > 10,000 primary DMC THAs, Darrith et al. [24] found an overall survivorship of 98.0% at a mean follow-up of 8.5 years. In previous studies consisting of high-risk patients treated with primary DMC THA, the survivorship has been as high as 97.4–98.0% with a follow-up ranging from 5.1 to 8.5 years [24, 25]. In a meta-analysis including 693 revision THAs from nine different studies, Levin et al. [16] found a survivorship of 94.5% for dual mobility with a mean follow-up of 31 months. In the present study including high-risk patients, the short-term survivorship was good and consistent with what has been reported previously. There was greater variation in demographics of the revision THA group compared with the primary THA group, which might explain the poorer survival and complications related to patients who underwent revision THA.

We found a low rate of dislocation (1.4%) after primary MDMC THA but a high rate (12.9%) after revision THA. A similar low dislocation rates (0.46–0.90%) after primary DMC and MDMC THAs have been reported in recent systematic reviews [24, 26]. Previous studies with similar implants used in revision THAs have shown lower dislocation rates varying from 2.2 to 11.0% [16, 27, 28]. Another treatment option for patients with a high risk of dislocation in addition to DMC is to use a liner with a locking mechanism. Van Ecke et al. [29] found in their meta-analysis that survivorship of primary DMC THAs were better than THAs with a locking mechanism (94.7% vs. 91.0%). Intraprosthetic dislocation is a rare complication related to an MDMC. In this study, it did not occur, while in previous studies the intraprosthetic dislocation rate was 1.13% in the primary group and 0.3–4.0% in the revision group [16, 24, 29, 30].

Complications other than dislocations were common in this high-risk cohort. The infection rate for the primary THA group was acceptable compared with similar studies [31, 32]. However, in revision cases, it was much higher than in a recent meta-analysis with a rate of 3.3% [16]. The patients in our study might have had more risk factors for infection, and the small number of patients in this group might have affected the results. In previous studies, the revision rates have been reported at 2.0%–3.4% after primary MDMC THAs and 3.4–13.5% after revision THAs, which are similar to our study [1, 7, 24, 33]. In this cohort, there were two (6.5%) re-revisions and one (3.2%) re–re-revision. All of them occurred in the revision group.

In this study, the 1-year mortality of high-risk THA patients after FNF was 10.0% which is similar to prior studies [34–36]. Recent literature supports current study findings as it has been shown that dislocation and mortality rates for FNFs treated with DMC THAs are comparable to other surgical options like unipolar and bipolar hemiarthroplasty [37]. Another recent meta-analysis compared the outcomes of patients that received DMC or hemiarthroplasty after FNF and found that patients who received MDMC had lower instability and mortality rates [38].

This study showed that implementation of MDMC at our institution have decreased annual hospitalization rate due to dislocation following THA almost 50%. These short-term results are promising and because of this finding, the use of MDMC has increased 25% in a short time at our institution. Based on these clinical findings, the use of MDMC seems to be a good option for high-risk hip fracture patients as it lowers risk for dislocation.

Long-term data are still lacking for MDMC implants, so surgeons should be aware of the potential benefits and pitfalls related to these implants [16]. The current study data were collected and analyzed in a more detailed way than in larger arthroplasty register studies which may help to find reasons and risk factors for implant failures more sensitive. Likewise, we considered the indications for surgeries in the analyses. We included all of these consecutive operations in a non-MDMC development institution, which reflects the learning curve and adaption of these implants. The limitations of this study include its retrospective nature, the small cohort size, and the short follow-up. A longer follow-up period and a larger cohort size are needed to observe late-occurring adverse events—for example, concerns regarding increased wear, intraprosthetic dislocation, and modular backside fretting corrosion.

### Conclusion

This retrospective cohort study of first 101 consecutively operated high-risk patients at our institution showed that primary MDMC has a low risk for postoperative dislocations and for other complications. Revision MDMC THAs had a high rate of complications and revisions, which reflects the complexity of surgery of these high-risk patients. Additional data will be needed to assess long-term survivorship of MDMC implants.

## Data Availability

Not available due to data protection reasons.

## Code availability

Not available due to data protection reasons.
